# A toolbox for generating multidimensional 3D objects with fine-controlled feature space: Quaddle 2.0

**DOI:** 10.3758/s13428-025-02736-w

**Published:** 2025-07-03

**Authors:** Xuan Wen, Leo Malchin, Thilo Womelsdorf

**Affiliations:** 1 Department of Psychology, Vanderbilt University, Nashville, TN 37240 USA; 2 Vanderbilt Brain Institute, Nashville, TN 37240 USA; 3 Department of Biomedical Engineering, Vanderbilt University, Nashville, TN 37240 USA

**Keywords:** Object recognition, Virtual reality, Augmented reality, Video games, Naturalistic stimuli, Psychophysical experiments

## Abstract

Multidimensional 3D-rendered objects are an important component of vision research and video-gaming applications, but it has remained challenging to parametrically control and efficiently generate those objects. Here, we describe a toolbox for controlling and efficiently generating 3D-rendered objects composed of 10 separate visual feature dimensions that can be fine-adjusted using Python scripts. The toolbox defines objects as multidimensional feature vectors with primary dimensions (object body related features), secondary dimensions (head related features), and accessory dimensions (including arms, ears, or beaks). The toolbox interfaces with the freely available Blender software to create objects. The toolbox makes it possible to gradually morph features of multiple feature dimensions, determine the desired feature similarity among objects, and automatize the generation of multiple objects in 3D object and 2D image formats. We document the use of multidimensional objects in a sequence learning task that embeds objects in a 3D-rendered augmented reality environment controlled by the gaming engine Unity. Together, the toolbox features enable the efficient generation of multidimensional objects with fine control of low-level features and higher-level object similarity useful for visual cognitive research and immersive visual environments.

**Supplementary Information:**

The online version contains supplementary material available at 10.3758/s13428-025-02736-w.

## Introduction

Investigating cognition requires tools that can precisely manipulate and control the features of stimuli presented to participants. Existing approaches have proposed multidimensional objects with a rich feature space including so-called geons (Biederman & Gerhardstein, 1993; Tarr et al., 1997), strings (Biederman & Gerhardstein, 1993; Edelman & Bülthoff, 1992; Tarr et al., 1997), Fribbles (Berry et al., 2015), amoeboids (Edelman & Bülthoff, 1992; Wong & Hayward, 2005), Greebles (Gauthier & Tarr, 1997), Ziggerins (Wong et al., 2009), YUFOs (Gauthier et al., 2003), Sheinbugs (Richler et al., 2017), and Quaddles (Watson et al., 2019), among other complex 3D-rendered object types (Cant & Goodale, 2007; Humphrey & Khan, 1992) (for examples, see Fig. 1 in Watson et al., 2019). This rich history of 3D-rendered multidimensional object sets illustrates that objects with a rich feature space have become indispensable in cognitive research for probing perception, recognition, and categorization processes (Arnott et al., 2008; Biederman & Gerhardstein, 1993; Bowman & Zeithamova, 2020; Chuang et al., 2012; Ghazizadeh et al., 2016; Knutson et al., 2012; Mercer & Duffy, 2015; Pusch et al., 2023; Todd, 2004; Wallraven et al., 2014; Wong et al., 2009). In previous research, Watson et al. (2019) introduced a toolbox that generated 3D-rendered objects with fine user control of up to five different visual feature dimensions (Watson et al., 2019). Multidimensional objects generated with this toolbox, so-called Quaddle objects, have been effectively utilized to explore various aspects of cognitive processing, such as object recognition, discrimination, visual search, attentional set shifting, and feature-value learning (Banaie Boroujeni et al., 2022; Boroujeni et al., 2022; Hassani et al., 2023; Kemp et al., 2024; Womelsdorf et al., 2021a, 2021b; Yazdanpanah et al., 2024). Moreover, 3D-rendered multidimensional objects are used in cognitive research using augmented reality (AR) and virtual reality (VR) that provide immersive environments with the aim of mimicking real-world experiences (Smith, 2019). For these dynamic and sometimes interactive settings, 3D rendering of objects can enhance task performance and has been used in contexts studying object identification, spatial manipulation and navigation, object memory recall, and learning (Corriveau Lecavalier et al., 2020; Howett et al., 2019; McIntire et al., 2014).

**Fig. 1:**
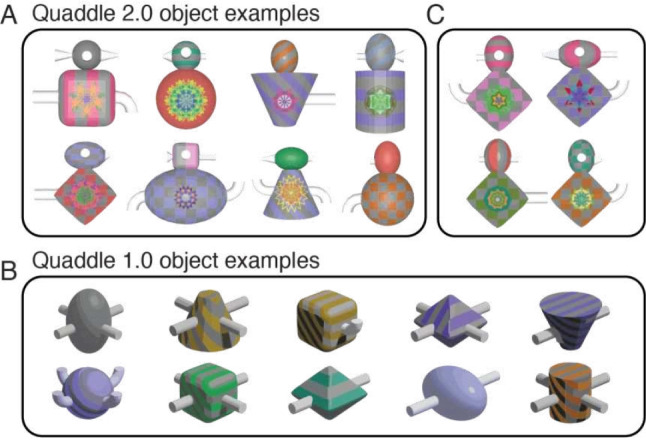
Examples of Quaddle objects. A Quaddle 2.0 objects varying in 10 different feature dimensions. **B** Simpler objects that vary in features of four dimensions (body shape, arm angle, body colors, body pattern), showing that the toolbox makes it possible to reproduce Quaddle 1.0 objects that were generated with a different software rendering suite (Studio DS Max). **C** Quaddle 2.0 objects that are more similar to each other by setting the similarity score > 8

Despite the usefulness of 3D-rendered objects in the described settings, researchers face challenges in generating well-controlled 3D objects due to technical constraints and the complexity associated with 3D modeling software. While various approaches have been employed to generate 3D stimuli for specific studies (Barry et al., 2014; Biederman & Gerhardstein, 1993; Gauthier & Tarr, 1997; Richler et al., 2017; Tarr et al., 1997; Wong & Hayward, 2005), it has been less common that software tools combine comprehensive parametric control over 3D object features with an easy-to-use interface for non-specialists. Here, we address this situation by introducing the Quaddle 2.0 toolbox, an evolution of the original Quaddle toolbox designed to enhance the utility and flexibility of object generation for dynamic visual and cognitive science studies and video-game contexts (Watson et al., 2019). Quaddle 2.0 expands upon its predecessor by increasing the available feature space (from 5 to 10 visual feature dimensions; Fig. 1A), while still allowing the generation of simpler Quaddle 1.0 objects that have fewer feature dimensions (Fig. 1B). In addition, the Quaddle 2.0 toolbox newly establishes the use of Blender—a free, open-source 3D creation suite compatible with macOS and Windows—and provides Python scripts that flexibly interface with Blender, allowing an efficient automatized control over low-level object features (gradual feature value changes) and higher-level 3D object characteristics (e.g., light viewing angle).

Here, we (i) detail the Quaddle 2.0 framework, (ii) introduce the scripting pipeline for generating large object sets, (iii) illustrate how users can control the feature similarity among objects, (iv) demonstrate the gradual morphing of multiple object features, and (v) showcase the use of Quaddle 2.0 objects in an experimental environment programmed with the Unity game engine to evaluate how nonhuman primates learn the sequential ordering of visual objects. Example code (in Python and MATLAB) and documentation with detailed instructions for using and customizing Quaddle 2.0 objects are freely available online (see Appendix).

## Method

### Composition of Quaddle 2.0 objects

**Fig. 2:**
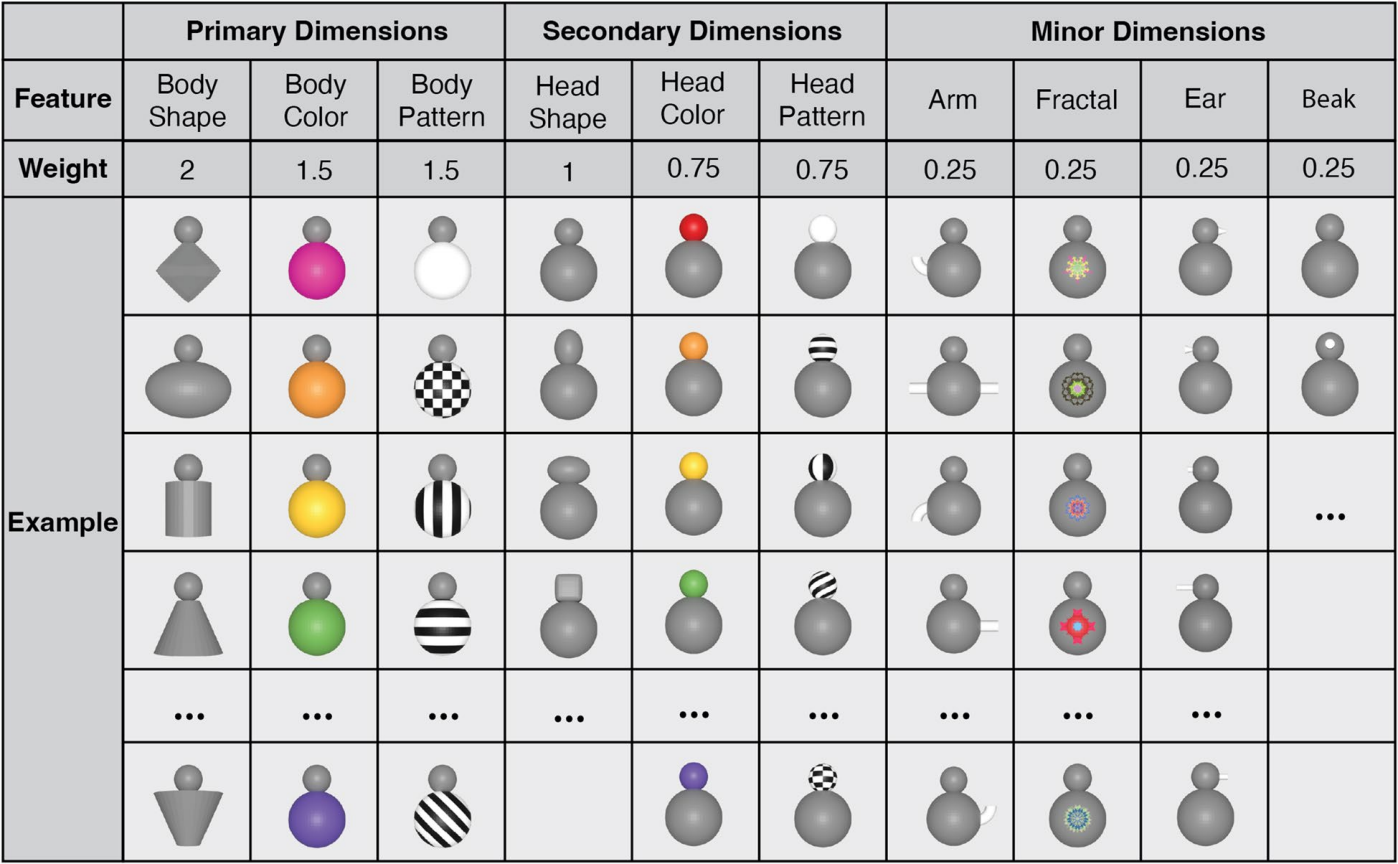
Object dimensions and example feature values pre-configured in the Quaddle 2.0 toolbox. Feature dimensions are categorized into primary dimensions (body-related), secondary dimensions (head-related), and minor dimensions (accessories), organized by size and front-view visibility. The ‘weight’ row denotes how a feature dimension is weighted when calculating the similarity of objects (*see* similarity score calculations)

Quaddle 2.0 objects vary in features of 10 preconfigured object dimensions (Fig. 2). The default object contains five main parts: head, body, arm, ear, and beak. The head and body can be covered with surface colors and patterns, and the body can have preconfigured, colorful fractal images projected on its center. The default arm, ear, and beak are colored white and can be adjusted to have different lengths, shapes, and bending angles. Arm, ear, and beak are considered minor feature dimensions that can be omitted from the objects (Fig. 2).

### Generating 3D objects using lists of preconfigured object features

**Fig. 3:**
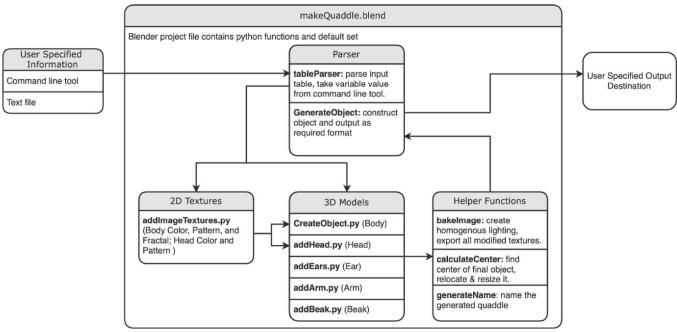
Generation pipeline for Quaddle 2.0 objects. The automated pipeline for generating Quaddle models using the *makeQuaddle.blend* Blender project file, which contains predefined Python functions. User-specified input (via either a command-line tool or a text file) is parsed by *tableParser.py*, which extracts the parameters used to generate the object with the script *GenerateObject.py*. The main pipeline splits into two key branches: (1) **2D Textures**, where functions apply body and head colors, patterns, and body fractals; and (2) **3D Models**, where scripts build the respective morphological elements. **Helper functions** handle lighting, object positioning, and naming before exporting to the user-specified destination

**Fig. 4:**
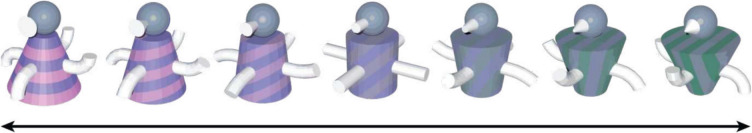
Illustration of gradual morphological changes between feature dimensions using the Quaddle 2.0 toolbox. The objects transition in body shape (from conical to cylindrical to upside-down conical), arm angles and orientations, color and surface pattern (from pink and purple horizontal stripes to green and blue vertical stripes), and beak shape (blunt to pointy)

Quaddle 2.0 objects are rendered using Blender (blender.org), a free and open-source 3D modeling software suite. Objects generated for figures in this article used Blender software version 3.4.0. The generation of Quaddle 2.0 objects follows a structured pipeline implemented through a series of Python scripts within a Blender project file as outlined in Fig. 3. All Python scripts are freely available online (see Appendix). The process begins with user-specified information about the desired feature dimensions and feature valuer per dimension, which are provided via a command-line tool or in a text file. The input is parsed using a parser function, which extracts the values for each visual feature of the different object dimensions. The core of the generation process involves several scripts that sequentially build the Quaddle object. Examples are surveyed in Fig. 2**.** Each component of the Quaddle is generated through a series of modular functions (Fig. 3). The process begins with the creation of the main body, followed by the attachment of the head. Subsequently, additional functions append the ears to the head, add arms to the body, and attach a beak to the head. This modular approach allows for precise control over each component of the Quaddle, enabling systematic manipulation of individual features while maintaining overall structural consistency. The body of the Quaddle object is initially generated from the default mesh shapes in Blender, including sphere, cube, cone, and cylinder, and then molded into the user-specified desired body shape. The arms, beak, and ears are initially generated as straight cylinders and molded into the desired angle, shape, and length. This arrangement allows the gradual morphing of individual object features by specifying intermediary values of bending angle, size of shape, and length. An example of objects that gradually morph four independent features is shown in Fig. 4. In the feature set preconfigured in the Quaddle 2.0 toolbox, each Quaddle has at most two arms, two ears, and one beak. There are three angles for the arm (bent up, bent down, straight), three shapes for the ear (straight, pointy, and blunt), and three lengths for the ear (regular, long, short). Different sub-features can be freely combined. Additional feature dimensions and feature values can be realized by modifying the preconfigured python code.

### Generating 2D textures and surface features

Following the structural assembly of the object, 2D textures are applied to the 3D model (Fig. 3). The script *addImageTextures.py* manages the application of body color, pattern, and fractal designs, as well as head color and pattern. The surface colors and patterns are imported from PNG files. The default gray color is the same for all objects, whereas the other colors are chosen within the CIE L*c* h* space such that the L* and c* values (luminance and saturation, respectively) are held constant, but h* values (hue) vary by 30°, meaning that there is a small difference in hue between the two colors, but not in the other components of the colors. The texturing process employs a UV project modifier, a specialized tool in Blender that projects 2D images onto the 3D object's surface. This modifier projects images from the front of the object, minimizing distortion when viewed frontally and ensuring symmetrical pattern application on both front and back. Two separate UV project modifiers are utilized for background patterns and surface fractals, each with a distinct projector object for optimal surface coverage. The process involves creating a material with a node-based shader network. Two image textures are incorporated and combined using a mixing operation, allowing for complex surface patterns. The first texture applies directly to the object surface, while the second serves as both a secondary color source and a blending mask. This approach prevents distortions of frontal views, facilitates symmetrical patterns, and allows control over texture placement and scale. By manipulating UV maps and projector properties, the process achieves various texture effects, from uniform coverage to complex, position-dependent patterns. The projection method can be similarly applied across different object geometries, which allows one to use the same complex visual pattern across a diverse set of object shapes.

The Quaddle 2.0 toolbox enables the projection of fractal images onto the object body or rendering various body surface patterns (Fig. 2). Fractal images are used because they provide a large and abstract image space that is unfamiliar to experimental participants. The fractals were generated following previously published methods (Ghazizadeh et al., 2016) using point-symmetrical polygons, where parameters such as size, edges, and color were randomly chosen to create distinct visual shapes. This method ensures that a large number of distinguishable fractals are available.

With regard to patterns for the body surface of objects, the Quaddle 2.0 toolbox has five preconfigured patterns available: *solid* (default), *horizontal stripes*, *diagonal stripes*, *vertical stripes*, and *grid*. Each pattern is generated as a 1,200 × 1,200 PNG file, with stripe widths set to 150 pixels to ensure the patterns and their associated colors remain clear and distinguishable in the final stimuli. While users can select any combination of colors for the patterns from the prepared asset folder, the current code defaults to combining a neutral gray with a user-specified secondary color (for examples, see Fig. 1). This design ensures that the colors are quite visible and have apparent contrast in the patterned image.

### Refinement and exporting of 3D-rendered objects

Post-processing steps are then executed to refine the generated Quaddle objects. These steps include creating homogeneous lighting conditions and exporting all modified textures, ensuring consistent visual quality across stimuli. The process also involves locating the center of the final object, repositioning and resizing it as necessary to maintain standardized dimensions across all generated Quaddle objects. Additionally, a unique identifier is assigned to each generated object, facilitating organized data management in subsequent experimental procedures.

The final stage constructs the complete object based on all specified parameters and outputs it in the required format to a user-defined destination. The Blender Python script can read definitions of multiple objects at once and create large sets of objects, exporting them either as image files (e.g., PNG) or 3D shape files (e.g., FBX, GLTF). The default output centers the object at the middle points of height and width of each object. The camera distance is adjusted based on the size of the objects in order to maintain a consistent apparent size across objects.

### Controlling the similarity of multidimensional objects

**Fig. 5:**
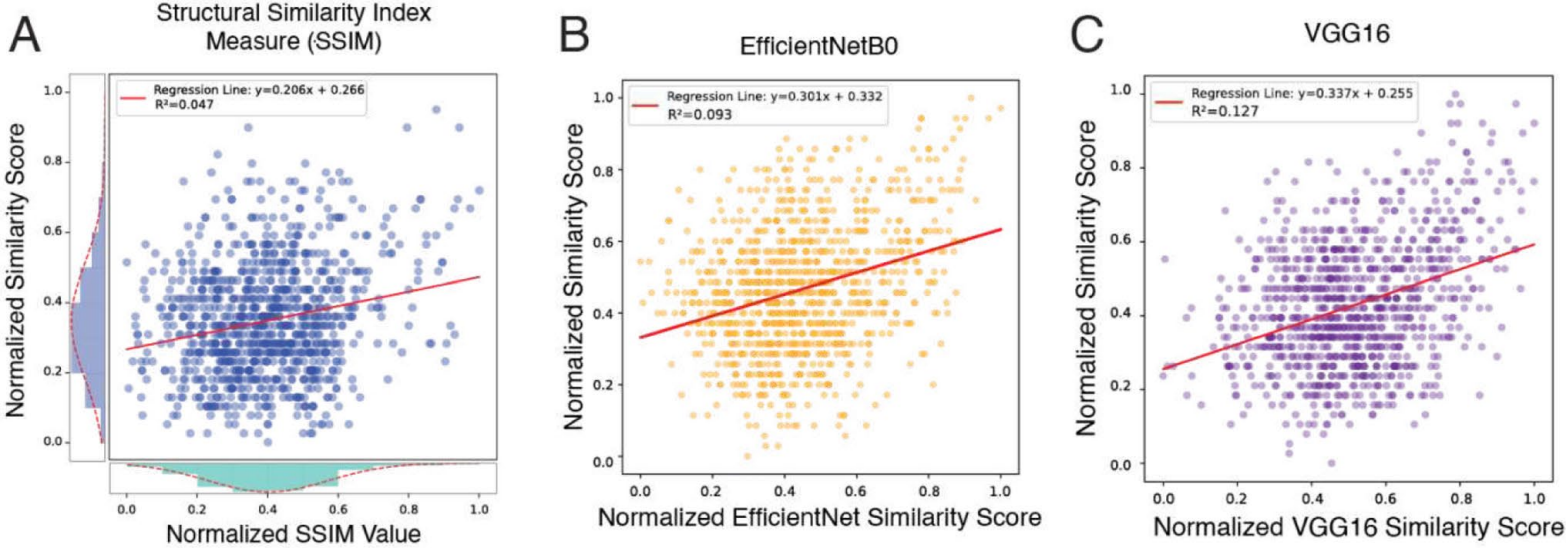
Comparison of object similarity scores. **A** Scatter plot showing the relationship between normalized SSIM values and similarity scores, including the distribution of both scores. The regression line is *y* = 0.206*x* + 0.266, with ${r}^{2}$= 0.047. **B** Scatter plot illustrating the correlation between normalized EfficientNetB0 similarity values and similarity scores. The regression line is *y* = 0.301*x* + 0.332, with ${r}^{2}$=0.093. **C** Scatter plot depicting the association between normalized VGG16 similarity values and similarity scores. The regression line is *y* = 0.337*x* + 0.255, with ${r}^{2}$= 0.127. Each figure is based on a random draw of 1,000 pairs of 300 randomly generated Quaddle images

**Fig. 6:**
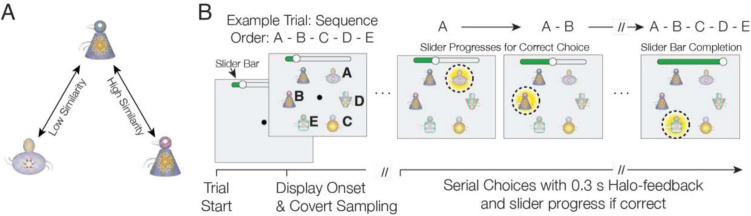
Experimental paradigm.** A** Illustration of Quaddle 2.0 objects that are perceptually dissimilar (left) and similar (right) as quantified by the similarity score (see text for details). **B** Each trial presented six objects. Monkeys learned to touch five objects in a predetermined order A-B-C-D-E and avoid a distractor object. A correct choice led to visual feedback (yellow halo) and increased the slider position at the top of the screen. Subjects had a maximum of 10 errors in exploring one sequence. Successfully completing one sequence led to fluid rewards. In each trial, the distractor object had higher similarity to either the second or fourth object in the sequence, compared with lower similarity to other objects

Quaddle 2.0 objects can share variable numbers of features, which we exploit by calculating a similarity score between objects. Users can set a similarity score in the *make_quaddle.m* MATLAB script when generating two or more objects. Similarity is calculated by comparing the values of each feature between two objects, with different weights assigned to each dimension (see Fig. 2). These weights are intended to reflect the presumed perceptual salience of each feature type, where visually dominant or structurally central features contribute more significantly to overall perceived similarity. Specifically, primary features like body shape (weight: 2), body color (weight: 1.5), and body pattern (weight: 1.5) are assigned higher weights due to their strong visual impact. Secondary features related to the head (head shape, head color, head pattern; all weighted 0.75) are considered moderately salient. Ancillary or less visually prominent features (arm, fractal, ears, beak; all weighted 0.25) receive lower weights, as they typically contribute less to the global perception of the object. This hierarchical weighting aims to produce similarity scores that better align with intuitive human judgments of object similarity. Using these feature dimension weights, the similarity score considers pairs of Quaddle objects ${{\varvec{Q}}}_{1}$ and${{\varvec{Q}}}_{2}$, defined by their dimension ${{\varvec{Q}}}_{1}=[{q}_{11},{q}_{12},\dots ,{q}_{1n}]$ and${{\varvec{Q}}}_{2}=[{q}_{21},{q}_{22},\dots ,{q}_{2n}]$. A binary vector indicates whether the values in each dimension are the same. This vector is defined as ${\varvec{B}}=[{b}_{1},{b}_{2},\dots ,{b}_{n}],$ where ${b}_{i}=1$ if${q}_{1i}={q}_{2i}$; otherwise,${b}_{i}=0$. Weights are predefined for each feature dimension$i$:${\varvec{W}}=[{w}_{1},{w}_{2},\dots ,{w}_{n}]$. The similarity score *S* is calculated as $S={\left({\varvec{W}}\right)}^{T}({\varvec{B}})$ or, equivalently, as $S={\sum }_{i=1}^{n}{w}_{i}{b}_{i}$. Using the preconfigured Quaddle 2.0 objects, the similarity score ranges from 0 (most dissimilar) to 11.75 (most similar). We validate the similarity score by comparing the similarity of object pairs as defined by the score with other quantitative similarity estimates (see below and Fig. 5) and by empirically evaluating the perceptual confusability of objects with high versus low similarity scores (see below and Fig. 6).

### Experimental paradigm for serial ordering of Quaddle objects with controlled feature similarity

To demonstrate a use case for the 3D-rendered Quaddle 2.0 objects, we generated multiple sets of Quaddle objects and trained four rhesus macaque monkeys to learn predefined temporal (serial) orders of five objects for each of those object sets in a sequence learning task. We defined the sixth object as a distractor with high similarity to only one of the other objects to illustrate that the distractor is confused specifically with an object that has high similarity to it as defined by the similarity score. All animal and experimental procedures used in this study complied with the National Institutes of Health Guide for the Care and Use of Laboratory Animals and the Society for Neuroscience Guidelines and Policies and were approved by the Vanderbilt University Institutional Animal Care and Use Committee.

The details of the experimental setup and the timing of task events are described in detail in Wen et al. (2025). In brief, four adult male rhesus monkeys performed the sequence learning task in their housing cage using cage-mounted touchscreen kiosk stations (Womelsdorf et al., 2021a, 2021b). Visual display, behavioral response registration, and reward delivery were controlled by the Multi-Task Universal Suite for Experiments (M-USE) (Watson et al., 2023). M-USE is a free software platform with preconfigured tasks (https://m-use.psy.vanderbilt.edu) that displays 3D-rendered objects using the Unity3D program (here: Unity version 2020.3.40F1). M-USE displayed the objects on an Elo 2094L 19.5″ LCD touchscreen with a refresh rate of 60 Hz and a resolution of 1,920 × 1,080 pixels, rendered at approximately 2.5 cm on the screen. Experimental sessions tested at least nine unique five-object (+ distractor) sequences, each with a unique set of Quaddle 2.0 objects. Sets of objects were generated by randomly assigning objects different features from up to 10 different feature dimensions (see Fig. 2). Five of the objects were defined to be dissimilar to make them easy to distinguish, while a sixth object was a distractor that was not part of a sequence but shared features with the object at either the second or the fourth serial order (Fig. 6A). The similarity of the distractor to selected objects of the sequence was used to confirm that the similarity of Quaddle 2.0 objects translated into perceptual confusability among objects with higher similarity.

As illustrated in Fig. 6B, the task presented on each trial six objects equidistant to each other at random locations on a circle equidistant from the center of the screen. Five of the objects were assigned a unique ordinal temporal position in the sequence of objects A-B-C-D-E, while a sixth object was a sequence-irrelevant distractor. In each trial, subjects chose objects by touching them and received either positive feedback (a yellow halo and high-pitched sound) for correct, or negative feedback (a transient gray halo and low-pitched sound) for choosing an object at an incorrect temporal position. After an erroneous choice, subjects had to re-choose the last correctly chosen object before making a choice of an object believed to be the next object in the sequence. Each trial allowed a maximum of 10 errors to complete the sequence and receive fluid reward. If the sequence was not completed, the objects were removed from the screen and a new trial was started with objects rearranged to new random locations. Subjects performed each sequence for a maximum of 15 trials to allow for the gradual acquisition of the sequential ordering and additional trials to exploit the learned sequence. For each correctly chosen object, the position of the slider of a progress bar at the top of the screen stepped forward. Successful completion of a sequence always completed the slider progress bar and resulted in a fluid reward. When a sequence was not completed, no fluid reward was given and the slider progress bar was reset for the subsequent trial with the same objects at new random locations. Choices of the distractor were registered as incorrect and not rewarded and required re-touching the last correctly chosen object.

### Hardware for automatic generation of Quaddle 2.0 objects

The Quaddle 2.0 toolbox was developed and tested on an Apple macOS system, but with minor adjustments it will be compatible with other operating systems that run Python and the Blender software. For the experiment, Quaddle 2.0 objects were generated with an Apple MacBook Pro 2023 with Apple M2 Max chip and 32 GB memory using MATLAB scripts (MATLAB 2022b) and Python version 3.10.8. The objects presented here were generated with Python scripts that connect with Blender version 3.4.0 (see Fig. 3). Links to comprehensive documentation and to the resources reproducing the objects are provided in the Appendix.

## Results

### Automatized generation of multidimensional objects

The Quaddle 2.0 toolbox connects the object creation software Blender with MATLAB via a command-line interface, enabling users to generate objects, bypassing Blender’s interface. This feature enables the automatized generation of large object databases with thousands of preconfigured objects. The MATLAB script *make_quaddle.m* (see Appendix) allows users to specify the number of objects, set the object similarity, and determine which features to keep or vary, providing high flexibility and utility for any application that requires multiple new objects across multiple cognitive experiments or augmented reality worlds. The script will generate an object table file for each specified object. A standard laptop is sufficient to generate objects. Objects displayed in Figs. 1, 2, 4, and 6 were generated on an Apple MacBook Pro (2023) equipped with the Apple M2 Max chip and 32 GB of memory.

### Computing efficiency for different formats

The preconfigured scripts (see Appendix) allow users to select any combination of 3D object formats (FBX, GLTF) or a 2D image output (PNG). The time taken to generate each object varies depending on the selected output format. When generating GLTF files only, the average time to generate and save an object was 1.32 s per object. For FBX files only, the time increased slightly, averaging 1.55 s per object. Generating PNG images took longer, with an average time of 2.35 s per object. When generating GLTF, FBX, and PNG files simultaneously, the time increased to 2.42 s per object, primarily due to the additional time required to render the 2D image compared to saving 3D object formats directly.

### Improvements of the Quaddle 2.0 platform over previous versions

Quaddle 2.0 objects provide more feature dimensions and a larger feature space than Quaddle 1.0 objects (Watson et al., 2019), but can be used with fewer than 10 feature dimensions, which enables the reproduction of objects with four feature dimensions similar to Quaddle 1.0 objects as shown in Fig. 1B. The Quaddle 1.0 framework had other limitations that the new framework addresses. Quaddle 1.0 objects were rendered and generated from within the Studio DS Max software on Windows PCs. By using the Blender software on PC or macOS operating systems and interfacing Python scripts to Blender, the Quaddle 2.0 platform moves beyond these limitations. This interface enables rapid “on-demand” generation of objects for applications that require multiple new objects with random combinations of features as is typical for multiple types of learning studies that introduce novel objects over hundreds of experimental sessions (Banaie Boroujeni et al., 2022; Treuting et al., 2024; Womelsdorf, Watson, et al., 2021).

### Gradual morphing between features of multiple dimensions

Category learning studies have demonstrated that fine, gradual manipulations of object features is needed for controlling the discriminability of relevant dimensions in cognitive tasks (Apostel & Rose, 2021; Folstein et al., 2012; Minda & Smith, 2001). The Quaddle 2.0 platform enables such fine control of low-level features and provides morphing functionality that can be applied to multiple features simultaneously as illustrated in Fig. 4. Gradual changes in feature values include morphing *body shapes*, adjusting the *angle of stripe patterns*, and modifying *color shading*. Additionally, Quaddle 2.0 offers precise control over *arm angle* and the *bluntness or sharpness of arm tips* (Fig. 4). Code and instructions for morphing specific colors and patterns are available on the website (see Appendix).

### Quantitative evaluation of the similarity score of objects

The MATLAB object generation script *make_quaddle.m* (see Appendix) accompanying the Quaddle 2.0 toolbox calculates the similarity among objects by determining the average number of features shared among objects in a given object set, weighting differently shared primary, secondary, and accessory features (see “Method”). We quantitatively evaluated this similarity measure by comparing it with two existing alternative similarity measures. First, we calculated the structural similarity index measure (SSIM), which measures the visual similarity between images by comparing luminance, contrast, and structure at the pixel level (Wang et al., 2004). We calculated the SSIM of Quaddle 2.0 objects exported as PNG images by applying the Python package *skimage*. We computed the SSIM and the similarity score for 1,000 pairs of object images randomly drawn from 300 randomly generated Quaddle 2.0 objects. Both the SSIM and the similarity score were moderately positively correlated with similar average SSIM and similarity scores (SSIM: mean = 0.4, variance = 0.03; similarity score: mean = 0.35, variance = 0.03; Fig. 5A). Secondly, we quantified how gradually increasing similarity scores of Quaddle 2.0 objects relate to a similarity score estimated by pretrained deep neural network models. These models process input data through multiple layers to extract meaningful patterns and features. At one or more hidden layers, the input data are transformed into dense vector representations known as feature embeddings. These embeddings are numerical outputs that capture the essential characteristics of images in a compact, multidimensional space (LeCun et al., 2015). We used the TensorFlow library (Abadi et al., 2016) to extract the feature vectors from the VGG16 model and the EfficientNetB0 model, which are pretrained on large datasets like ImageNet (Simonyan & Zisserman, 2014; Tan & Le, 2019). Feature embedding vectors of two images were extracted and cosine similarity between two vectors was calculated as the similarity score. The networks allow users to choose similarity metrics based on low-level structural comparisons or evaluation of high-level feature similarity. We found that the similarity score shows significant albeit moderately positive correlations with the EfficientNetB0 similarity value, with an ${r}^{2}$ value of 0.09 (slope = 0.301; intercept = 0.332) (Fig. 5B). Similarly, the VGG16 similarity value was also moderately correlated, with an ${r}^{2}$ value of 0.127 (slope = 0.337; intercept = 0.255; Fig. 5C). These results show that scoring the similarity of Quaddle 2.0 objects based on shared feature spaces with a differential weighting of primary, secondary, and minor feature dimensions is weakly but positively correlated with quantitative assessments of low-level feature similarity metrics.

### Empirical evaluation of Quaddle 2.0 objects and their similarity score

**Fig. 7:**
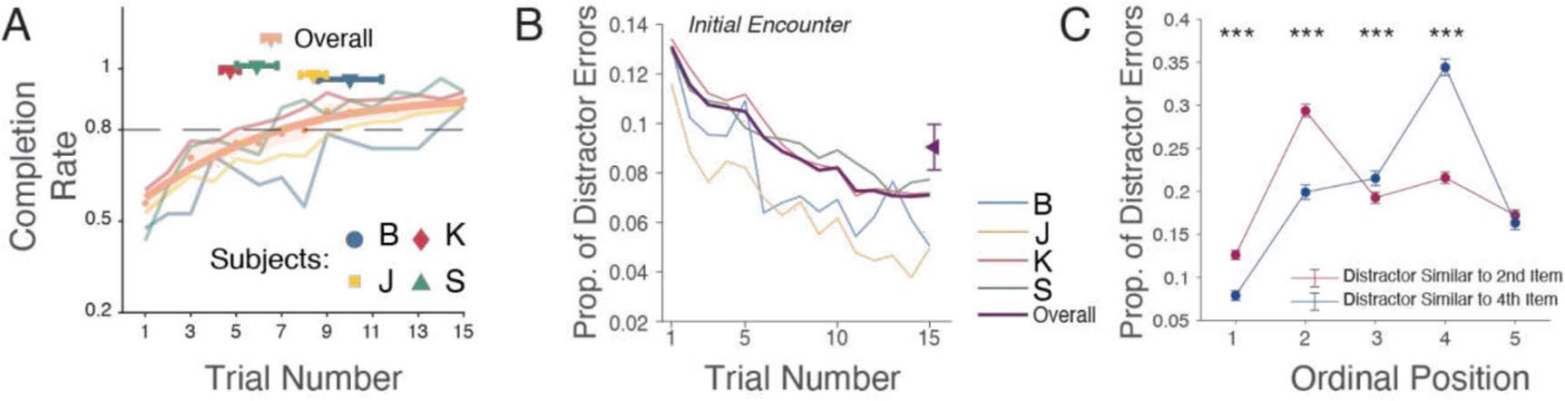
Experimental results.** A** Learning curves for completing sequences with ≤ 10 errors (*y*-axis). Symbols show avg. trial and SE to reach 80% completion for each subject and overall. **B** Proportion of distractor choices across trials. The mean value is shown as the rightmost data point (mean ± 95% CI, 0.090 ± 0.010). **C** Distractor choices at each ordinal position when the distractor is similar to the object in the second ordinal position and when the distractor is similar to the object in the fourth ordinal position. Two-proportion *Z*-tests were applied at each ordinal position for comparing the difference. Stars denote the significance level. (Ordinal position: before and after swap, mean ± 95% CI) 1: 0.13 ± 0.01; 1: 0.08 ± 0.01; 2: 0.29 ± 0.01; 2: 0.20 ± 0.01; 3: 0.19 ± 0.01; 3: 0.22 ± 0.01; 4: 0.22 ± 0.01; 4: 0.34 ± 0.01; 5: 0.17 ± 0.01; 5: 0.16 ± 0.01. Figure panels **B** and **C** were adapted from Wen et al. (2025)

To test how subjects learn to associate different Quaddle 2.0 objects that are dissimilar and to test how higher similarity scores correspond to perceptual confusability of objects, we tested rhesus monkeys on a sequence learning task. The testing was part of a larger empirical study (Wen et al., 2025) and included 125 testing sessions across four adult male rhesus monkeys (subject B = 13, subject J = 40, subject K = 59, subject S = 13). Each session contained on average 24 (range = 9–26) sequence learning blocks. For each of the learning blocks in each of the sessions, six new Quaddle 2.0 objects were generated (five objects that were part of the sequence, and one distractor object). Subjects learned the predetermined five-object sequences. They reached criterion performance (completing a sequence with maximally 15 choices, i.e., 10 or fewer erroneous choices) within 6.53 ± 0.36 trials (subject B = 10.00 ± 1.39; J = 8.39 ± 0.58; K = 4.74 ± 0.40; S = 5.91 ± 0.89; Fig. 7A). Learning was accompanied by a steady decrease in the erroneous choices of the distractor (Fig. 7B). In the experiment, the distractor object resembled either the second or fourth object of the sequence, and distractor errors occurred significantly more often at positions at which the sequence-relevant object and the distractor were similar, i.e., the distractor object was more easily confused with objects that had higher similarity scores (Fig. 7C).

## Discussion

We introduced the Quaddle 2.0 toolbox for generating 3D-rendered objects with multiple controllable feature dimensions for cognitive research and video-gaming applications. Building on the foundation of the original Quaddle 1.0 framework, Quaddle 2.0 enhances stimulus generation by expanding the number of independently varying feature dimensions and improving the script-based accessibility through the use of Blender, an open-source 3D modeling platform with Python support. Quaddle 2.0 allows the use of simple configuration text files to define objects and fine adjustment of low-level object features, which makes it easier to create custom object sets and automatize the generation of large object sets. Quaddle 2.0 supports various 3D model formats, which enables their use in augmented and virtual reality platforms. Together, these toolbox features facilitate dynamic and immersive experimental designs.

It can replicate the key features from the original Quaddle framework (Watson et al., 2019), which has been successfully tested in experiments with both humans and nonhuman primates (Banaie Boroujeni et al., 2022; Boroujeni et al., 2022; Hassani et al., 2023; Kemp et al., 2024; Womelsdorf et al., 2021a, 2021b; Yazdanpanah et al., 2024). Extending this earlier framework, we have shown how the Quaddle 2.0 was used in a sequence learning experiment that required hundreds of new, unique objects in each experimental session, and which controlled the similarity among objects. Non-human primates learned to discriminate and serially order the objects and were confused by distractors when they shared high feature similarity with target objects (Fig. 6). Taken together, the results of this example use case suggest that Quaddle 2.0 is a versatile, scalable, and user-friendly tool that enables cognitive research in 3D-rendered environments.

**Fig. 8:**
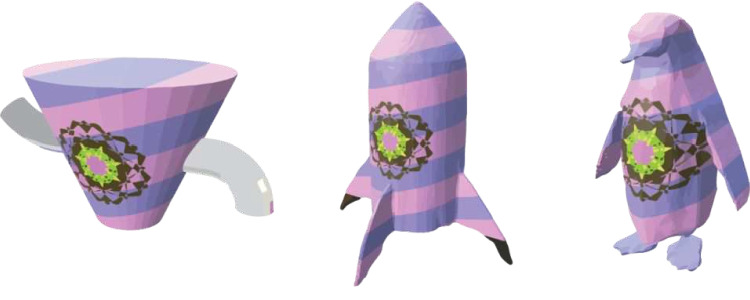
Possible Quaddle objects. Quaddle 2.0 object, rocket, and penguin covered with the same color, pattern, and fractal on their bodies. Rocket and penguin were generated by Shap-E, a text-to-3D model

Looking ahead, while Quaddle 2.0 already offers substantial flexibility for expanding stimulus diversity through the modification of part composition, as we have demonstrated, future advancements could further revolutionize the generation of unique 3D objects. A particularly promising avenue involves the integration of sophisticated generative diffusion models directly into the stimulus creation pipeline. We have experimented with current text-to-3D models, such as Shap-E (https://huggingface.co/spaces/hysts/Shap-E), to generate base forms that can then be customized within the Quaddle 2.0 framework (Fig. 8). While these initial explorations are encouraging, the ideal future direction would involve a stable, fast, and locally runnable diffusion model capable of generating high-quality 3D meshes from text prompts or other inputs. Such a model, when combined with the parametric control and Blender scripting capabilities of Quaddle 2.0, could unlock the potential to create a virtually limitless array of novel 3D objects, tailored precisely to experimental needs. This would allow researchers to rapidly prototype and generate stimuli representing highly diverse categories, from specific animals and tools to abstract forms, far beyond the current default Quaddle structure or easily sourced external models.

## Electronic supplementary material

Below is the link to the electronic supplementary material.

Supplementary file1 (XLSX 555 KB)

## Data Availability

Not applicable.

## Appendix

- Python scripts and installation instructions for the pipeline generating Quaddle 2.0 objects (shown in Fig. 3) are available at https://github.com/xwen1765/blender-quaddle. Setting up the toolbox involves three steps: (1) Download Blender 3.4.0 and Python code from the GitHub repository on the local computer. (2) Open Blender software, change preference as in the instruction (see below: *installation guide*). (3) Open the file *makeQuaddle.blend* and run the script parser.py; an example Quaddle will be created and can be viewed in Blender. To automatically generate object tables and generate 3D object sets with user-defined similarity between objects, the user can use MATLAB. The GitHub repository includes the files *make_quaddle.m* and *test_example.m* (https://github.com/xwen1765/blender-quaddle/Matlab) that show how the configuration variables are set, object table files are generated, and the parser.py is called to render and generate the Quaddle 2.0 objects.
- A website installation guide is available on https://xwen1765.github.io/posts/Quaddle/; video instruction is on https://www.youtube.com/watch?v=FOaKS-hQfYI.
- A website documentation about MATLAB scripts is available in the text file “*Documentation-MATLAB-make_quaddle.pdf*” on the website https://m-use.psy.vanderbilt.edu/quaddles-2/ and on the GitHub repository: https://github.com/xwen1765/blender-quaddle/Matlab/Documentation-MATLAB-make_quaddle.pdf
- An example of the morphing functionality is included in the main.py script on the GitHub repository. The script can be run directly to generate an example Quaddle in Blender. Users can specify parameters such as arm angle, body shape ratio, body pattern color, stripe angle, and beak size within the first few lines of the main function. Additionally, users can use the same function to add more arms (front and back) and other accessories beyond the default Quaddle feature set. To morph the pattern and color on the body, a Python script for generating pattern images is provided on the GitHub repository: https://github.com/xwen1765/blender-quaddle/PatternGeneration

## Code availability

All MATLAB and Python scripts of the Quaddle 2.0 toolbox are publicly available via https://github.com/xwen1765/blender-quaddle

## Declarations

### Open practices statement

The data and materials for all experiments are available at the GitHub repository, and none of the experiments was preregistered.
